# Effects of dehydroepiandrosterone alone or in combination with a high-fat diet and antibiotic cocktail on the heterogeneous phenotypes of PCOS mouse models by regulating gut microbiota

**DOI:** 10.3389/fendo.2022.1030151

**Published:** 2022-12-22

**Authors:** Xuejiao Wang, Liping Gu, Yahui Zhang, Chuanhao Xiong, Yongde Peng, Xiaoying Ding

**Affiliations:** Department of Endocrinology and Metabolism, Shanghai General Hospital, Shanghai Jiao Tong University School of Medicine, Shanghai, China

**Keywords:** polycystic ovary syndrome, mouse model, phenotype, dehydroepiandrosterone, high-fat diet, antibiotic cocktail

## Abstract

**Objective:**

Polycystic ovary syndrome (PCOS) is a heterogeneous endocrine and metabolic disease. The gut microbiota is highly correlated with androgen secretion and insulin resistance (IR), which are two potential major pathogenic mechanisms of PCOS. Currently, an antibiotic cocktail (ABX) is often used to construct pseudo germ-free mouse models for studies on the gut microbiota and PCOS. Our work aimed to study the effects of dehydroepiandrosterone (DHEA), a high-fat diet (HFD) and ABX on the heterogeneous phenotypes of PCOS mouse models by regulating the gut microbiota.

**Methods:**

PCOS mouse models were established by subcutaneous injection of DHEA alone or in combination with a HFD in wild-type and pseudo germ-free mice. The changes in ovary morphology and sex hormonal and glycolipid metabolic parameters were evaluated.

**Results:**

Wild-type mice treated with DHEA or DHEA+HFD showed a PCOS-like phenotype of hyperandrogenism, anovulation and polycystic ovaries. The former was combined with hyperinsulinemia and IR, while the latter was combined with glucolipid metabolic disorders, extremely heterogeneous hyperinsulinemia and IR. The phenotype of PCOS mice, especially the metabolic parameters, was correlated with the gut microbiota. The pseudo germ-free mice treated with DHEA or DHEA+HFD also showed a PCOS-like phenotype. However, DHEA could not induce hyperinsulinemia or IR in pseudo germ-free mice. Pseudo germ-free mice treated with DHEA+HFD exhibited decreased serum AMH level, glucolipid metabolic disorders and IR. Compared with the wild-type mice, the pseudo germ-free mice treated with DHEA showed significantly higher testosterone and lipid levels and lower blood glucose levels, and they did not present with hyperinsulinemia or IR.

**Conclusion:**

A better and stabilized mouse model simulating the pathophysiological defects of PCOS was induced by DHEA alone rather than by DHEA+HFD. The ABX intervention improved glucose metabolic disorders and hyperinsulinemia but aggravated the hyperandrogenism and lipid metabolic disorders of the PCOS mice. This study suggests that the gut microbiota plays an important role in the heterogeneous phenotypes of PCOS mouse models.

## Introduction

Polycystic ovary syndrome (PCOS) is a common, complex, and heterogeneous endocrine and metabolic disorder that affects 8 to 13% of women of reproductive age and 21% of women in high-risk groups (1). Based on the Rotterdam criteria (2), PCOS can be identified into 4 phenotypes/subtypes, and the presence of insulin resistance (IR) and hyperinsulinemia is common among women with PCOS but are not required for diagnosis (3). Despite significant progress in understanding this disorder over the past 20 years, the heterogeneity of PCOS makes the pathophysiology and treatment still confused. Because of ethical and logistical constraints on clinical studies including women with PCOS, many studies need to be carried out in animal models, especially rodent models.

To date, there are no accepted rodent models of PCOS to comprehensively simulate the pathophysiological process and phenotypes of women with PCOS. Rodent models of PCOS induced by dehydroepiandrosterone (DHEA), dihydrotestosterone or letrozole are widely used in most studies (4–8). These models share the key characteristics of women with PCOS, including hyperandrogenism, disrupted cyclicity, presence of follicular cysts, and some metabolic disorders. However, the above models display high heterogeneity mainly in the presence of metabolic disorders including IR and hyperinsulinemia (9, 10), which leads to difficulties in the repeatability and reliability of further PCOS animal studies. Lai et al. (11) firstly induced a PCOS mice model with distinct metabolic features *via* DHEA combined with a high-fat diet (HFD), confirming the effect of HFD on the heterogeneous phenotypes of PCOS mice.

Studies suggest that the gut microbiota is involved in the genesis of PCOS. In 2012, Tremellen and Pearce proposed a hypothesis that dysbiosis of the gut microbiota is a causative factor of metabolic and reproductive manifestations of PCOS (12). In the past 10 years, studies in both humans and rodent models have demonstrated that alterations of the gut microbiota are associated with PCOS (7, 8, 13, 14). Most evidence has focused on the connection between the gut microbiota and hyperandrogenism and IR, which are the two core pathogenic factors of PCOS. Torres et al. reported a relationship between the gut microbiota and hyperandrogenism, although the genesis is still unclear (15). However, the gut microbiota could cause IR/hyperinsulinemia *via* the immune system and microbiota-gut-brain axis (13, 16, 17). The above evidence proves that the gut microbiota participates in the two core pathogenic factors of PCOS *via* different pathways, suggesting that the gut microbiota may be related to the heterogeneity of PCOS. Our previous study found that the gut microbiota was closely related to the heterogeneous phenotypes of women with PCOS (18). However, the correlation between the gut microbiota and the heterogeneity of PCOS rodent models has not yet been studied.

Based on previous studies of PCOS mouse models, this study aimed to observe whether the gut microbiota plays a role in producing the heterogeneity of PCOS mouse models. We induced PCOS in different mouse models using an injection of DHEA or an injection of DHEA combined with a HFD in wild-type mice and pseudo germ-free mice to observe the effects of the gut microbiota on the phenotypes of PCOS mouse models.

## Materials and methods

### Animals

Eighty-four female C57BL/6J mice (3 weeks old) were purchased from Shanghai Laboratory Animal Center (SLAC). Five mice per cage were housed in a specific pathogen-free environment at the Shanghai Jiao Tong University animal center under a standard temperature (22°C ± 3°C), stable humidity, and standard lighting conditions (12 h light/12 h dark cycle), with 24 h free access to irradiated rodent feed and autoclaved water. Body weight was measured weekly.

### Establishment of a PCOS model without gut microbiota depletion

Forty mice (6 weeks old) were randomly divided into four groups: the Ctrl, HFD, DHEA and DHEA+HFD groups (n = 10 each group). To induce the PCOS model, the mice in the DHEA and DHEA+HFD groups were given a daily subcutaneous injection of DHEA (6 mg/100 g body weight; D4000-10 g, Sigma Aldrich, USA) dissolved in 0.1 mL of sesame oil for 5 weeks. For the Ctrl group, the mice were fed a normal diet and injected daily with sesame oil. For the HFD group, the mice were fed a HFD (60% of energy provided by fat, D12492, Research Diets, USA) and injected daily with sesame oil. For the DHEA group, the mice were fed a normal diet and injected daily with DHEA. For the DHEA + HFD group, the mice were fed a HFD and injected daily with DHEA.

### Establishment of a PCOS model with gut microbiota depletion

To deplete the gut microbiota, forty-four mice (3 weeks old) received a freshly prepared antibiotic cocktail (ABX) in their drinking water that was prepared with ampicillin (1 g/L, A105483), vancomycin (500 mg/L, V105495), metronidazole (1 g/L, M109874), and ciprofloxacin (200 mg/L, C131636) (all from Aladdin, China). After 3 weeks of ABX treatment, thirty-one mice (6 weeks old) were randomly divided into four groups: the ABX group (n = 10), HFD+ABX group (n = 5), DHEA+ABX group (n = 8) and DHEA+HFD+ABX group (n = 8). The mice were injected and fed in the same manner as the mice without gut microbiota depletion and continued to receive ABX water for another 5 weeks.

### Oral glucose tolerance test

An oral glucose tolerance test (OGTT) was performed 4 weeks after starting the DHEA treatment. The mice were fasted overnight for 6 hours before the OGTT. The glucose level of blood from the tail vein was measured with a glucometer (Accu-Chek^®^ Performa) before and 15, 30, 60, 90 and 120 min after the administration of glucose (2 g/kg body weight per mL) by oral gavage. Tail vein blood was also collected for serum insulin measurement at 0-, 15-, and 60-min time points. The area under the curve of the glucose levels (AUC-Glucose) was calculated using GraphPad Prism 6.0 software.

### Vaginal smears and oestrous cycle determination

Vaginal smears were taken daily at 8:00–9:00 am for 2 weeks before the OGTT to avoid the potential disturbances caused by the OGTT. Vaginal smears were stained with Giemsa for light microscopic determination of oestrous cycle stage. The predominance of nucleated epithelial cells indicated the proestrus stage. The predominance of cornified squamous epithelial cells indicated the oestrus stage. When the number of cornified squamous epithelial cells decreased as numerous white blood cells and nucleated epithelial cells appeared, the mice were in the metoestrus stage. Thin vaginal mucosa with vaginal cells comprising nearly all white blood cells indicated the dioestrus stage. It is worth noting that samples from mice treated with DHEA were all cornified squamous epithelial cells throughout the experiment.

### Morphology

After the collection of blood samples, the ovaries were removed and fixed in 4% paraformaldehyde immediately. The tissues were then embedded in paraffin and stained with haematoxylin and eosin (HE). Corpora lutea and antral follicles were counted from the middle section of each ovary. Antral follicles displayed an expanded antral cavity, collapsed walls, and an attenuated and scattered granulosa cell layer.

### Serum measurement

At the end of the experiment, blood for the determinations of testosterone (T) and lipid levels was collected from the inner canthus after the mice fasted overnight for 6 hours. Levels of serum T were measured using ^125^I-labelled radioimmunoassay kits (B10B, Beijing North Institute of Biological Technology, China). The within-assay and between-assay variabilities were 10% and 15%, respectively. Levels of serum lipids were measured using assay kits according to the manufacturer’s instructions for the following lipids: triglycerides (TGs; A110-1), total cholesterol (TC, A111-1), high-density lipoprotein cholesterol (HDL-C; A112-1) and low-density lipoprotein cholesterol (LDL-C; A113-1) (all from Nanjing Jiancheng, China).

Levels of serum insulin were measured using an ultrasensitive mouse insulin ELISA kit (90080, Crystal Chem, USA). The area under the curve of the insulin levels (AUC-Insulin) was calculated using GraphPad Prism 6.0 software. HOMA-IR = fasting blood glucose (FBG) (mmol/L) ∗ fasting blood insulin (FINS) (mIU/L)/22.5. Serum anti-Mullerian hormone (AMH) levels were measured using a mouse AMH ELISA kit (YLK-EXS921, Youlike Life Science, China).

### Fecal sample collection and 16S rRNA gene sequencing

Fresh fecal samples were collected before sacrifice and were immediately stored at −80°C until analysis. DNA extraction from frozen fecal samples was conducted as previously described (19). All DNA samples were sequenced with a MiSeq reagent kit v3 (600-cycle) (MS-102-3033, Illumina, USA). A sequencing library for the V3–V4 regions of the bacterial 16S rRNA genes was constructed based on the manufacturer’s instructions, with some modifications as previously described (20).

### Microbiota data analysis

The 16S rRNA gene sequence data were processed and analysed using QIIME2 software (v2018.11) (21). The raw sequence data were demultiplexed and then denoised with the DADA2 pipeline (q2-dada2 plugin) (22) to obtain the amplicon sequence variant (ASV) frequency data table. Alpha diversity metric (observed ASVs), beta diversity metric (Bray−Curtis distance), and principal coordinate analysis (PCoA) were performed using the q2-diversity after rarefying the samples to 11,000 sequences per sample. Taxonomic assignment for ASVs was performed *via* the q2-feature classifier (23) using the SILVA rRNA gene database (24). The observed ASV index was compared using the Kruskal–Wallis test. The treatment-induced structural shifts in the gut microbiota were evaluated using Bray−Curtis distance, visualized by PCoA plot, and assessed by permutation multivariate analysis of variance (PERMANOVA) using the R “vegan” package with 9,999 permutations.

ASVs shared by at least 25% of all samples were considered prevalent ASVs. The correlation coefficients between the ASVs were calculated by the SparCC algorithm (25). The correlations were converted to a correlation distance (1- correlation coefficients) and then clustered into 59 coabundant groups (CAGs) using Ward clustering and PERMANOVA with 9,999 permutations. Spearman correlations between CAGs and parameters were calculated by MATLAB R2014a, and the Benjamini−Hochberg procedure was used to control the false discovery rate.

### Statistical analysis

Statistical analysis and data visualization were performed using GraphPad Prism 6.0 software. Data are presented as the means ± SEMs. One-way analysis of variance (ANOVA) followed by the correction of *p* values with the Tukey post test was used to assess the differences among four groups (i.e., Ctrl, HFD, DHEA and DHEA+HFD or ABX, HFD+ABX, DHEA+ABX and DHEA+HFD+ABX). A two-tailed unpaired Student’s t test was used to compare the differences between the two groups (i.e., Ctrl vs. DHEA, ABX vs. DHEA+ABX, Ctrl vs. ABX, and DHEA vs. DHEA+ABX). A difference with a *p* value < 0.05 was considered statistically significant.

## Results

### Different treatments induced a PCOS-like phenotype in wild-type mice

**Figure 1:**
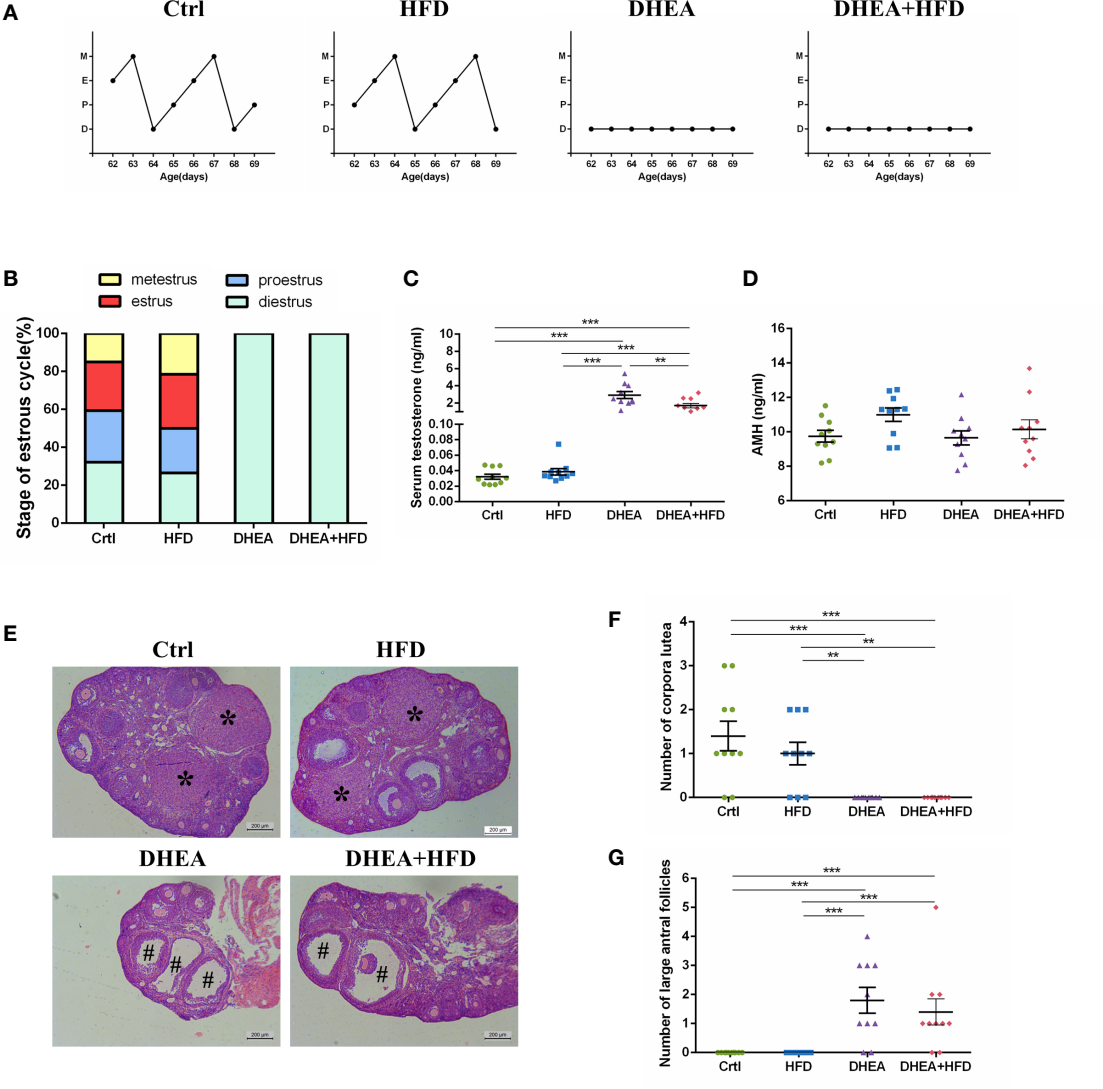
The stage of the oestrous cycle, ovarian morphology and serum T level in wild-type mice. **(A)** Representative oestrous cycle of one mouse from each group. **(B)** The proportion of each oestrous cycle stage in each group. **(C)** Serum testosterone level. **(D)** Serum AMH level. **(E)** Representative HE staining of ovarian tissue from one mouse from each group. * indicates corpora lutea, and # indicates large antral follicles. The number of **(F)** corpora lutea and **(G)** large antral follicles of one ovary from each group. Data are reported as the means ± SEMs. **P <0.01, and ***P < 0.001. AMH, anti-Mullerian hormone.

All of the mice from the DHEA and DHEA + HFD groups stayed in the dioestrus stage (**Figures 1A, B**), and the serum T levels were significantly higher in these mice than in mice from the Ctrl and HFD groups (**Figure 1C**). There was no significant difference in the AMH level among the four groups (**Figure 1D**). The T level was significantly higher in the DHEA group than in the DHEA+HFD group. Ovaries from the Ctrl and HFD groups contained follicles at different stages of development and several corpora lutea (CL). In contrast, those from the DHEA and DHEA + HFD groups contained several antral follicles and no CL (**Figures 1E–G**). These results verified the successful establishment of a PCOS-like mouse model with hyperandrogenism, anovulation and polycystic ovaries.

**Figure 2:**
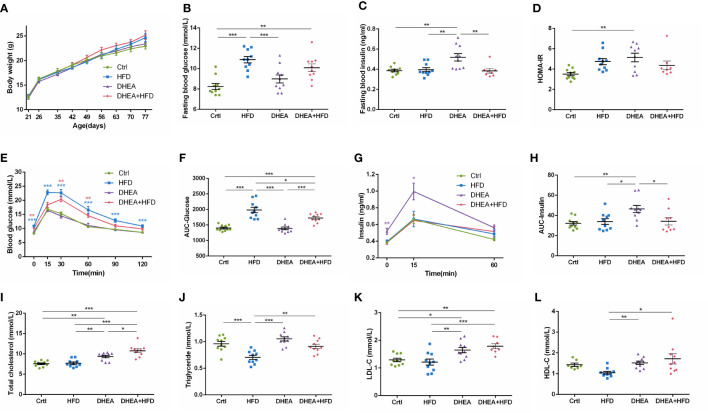
Glucolipid metabolism in wild-type mice. **(A)** Body weight. **(B)** Fasting blood glucose levels. **(C)** Fasting blood insulin levels. **(D)** HOMA-IR. **(E)** Serum glucose levels during the OGTT. **(F)** AUC-Glucose. **(G)** Serum insulin levels during the OGTT. **(H)** AUC-Insulin. **(I)** Total cholesterol. **(J)** Triglycerides. **(K)** LDL-C. **(L)** HDL-C. *P < 0.05, **P <0.01, and ***P < 0.001. HOMA-IR, homeostasis model assessment for insulin resistance index; AUC, area under the curve; LDL-C, low-density lipoprotein cholesterol; HDL-C, high-density lipoprotein cholesterol.

Body weights were similar among all of the groups (**Figure 2A**). Compared with the Ctrl and DHEA groups, the HFD and DHEA+HFD groups displayed markedly higher FBG levels (**Figure 2B**) and glucose intolerance (**Figures 2E, F**). Two mice from the DHEA+HFD group showed extremely heterogeneous hyperinsulinemia and IR. Thus, only the DHEA group displayed significant and stabilized increases in FINS, HOMA-IR and postprandial insulin levels (**Figures 2C, D, G, H**), indicating hyperinsulinemia and insulin resistance in the DHEA group. In addition, the DHEA and DHEA + HFD groups displayed significantly increased TC and LDL-C levels compared with the other groups, while the mice on a HFD displayed a reduced trend for TG levels (**Figures 2I–L**).

**Figure 3:**
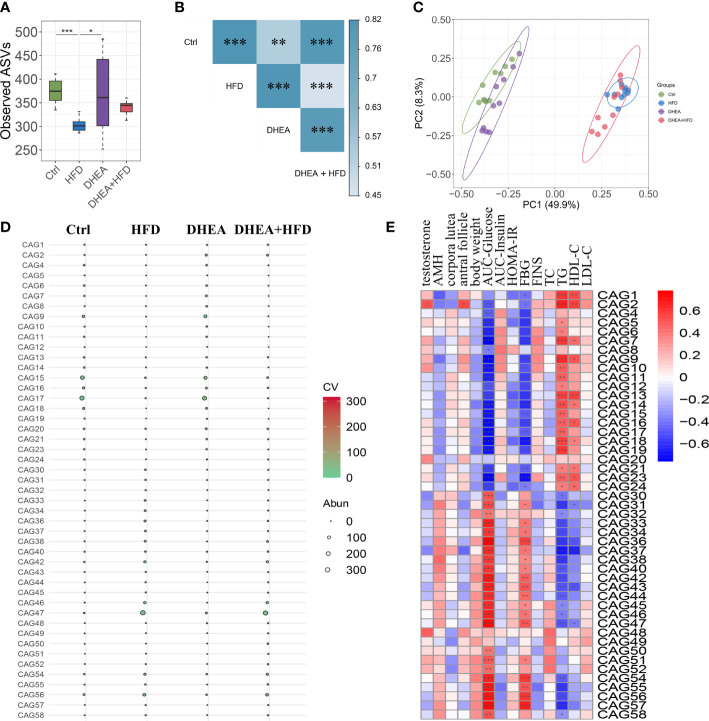
The gut microbial structure was altered in PCOS mice. Alpha diversity measured by **(A)** observed ASVs. Data are expressed as the mean ± SEM. *P < 0.05 and ***P < 0.001. **(B)** A PERMANOVA test was performed on the basis of the Bray−Curtis distances. The colors of the blocks indicate the distance, and the asterisks denote significant differences between different groups. ** *P <*0.01 and *** *P* < 0.001. **(C)** Principal coordinates analysis (PCoA) performed on the basis of the Bray−Curtis distances. PC1, principal coordinate 1; PC2, principal coordinate 2. **(D)** Bubble plot shows the variation in the average abundance of CAGs in each group. The size and color of the circles represent the average abundance and coefficient of variance (CV) of each CAG, respectively. **(E)** Correlations between phenotypic data and CAGs.

We performed V3-V4 16S rRNA gene sequencing to compare the diversity and composition of the gut microbiota of the four groups. The observed ASVs showed a significant reduction in alpha diversity in the HFD group compared to the Ctrl and DHEA groups, but no significant difference was observed among the Ctrl, DHEA and DHEA+HFD groups (**Figure 3A**). In the context of beta diversity based on Bray–Curtis distance (**Figures 3B, C**), the overall microbial structure showed significant differences among the four groups, but the HFD might contribute to a more severe effect. We constructed a coabundance network between the 520 ASVs that were shared by at least 25% of all samples and clustered the ASVs into 59 coabundant groups (CAGs) (**Table S1**). Of these, CAG2, mainly containing ASVs from *Bifidobacterium* and *Lactobacillus*, was significantly increased in the DHEA and DHEA+HFD groups. CAG2 was also positively correlated with the T level, the number of antral follicles and the TG level and negatively correlated with the AUC-Glucose and FBG levels. CAG6 composed of ASVs from *Alistipes* and *Turicibacter*, CAG9, CAG15 and CAG17 composed of ASVs from *Muribaculaceae*, CAG16 composed of ASVs from *Lactobacillus* and *Muribaculaceae*, and CAG18 composed of ASVs from *Lachnospiraceae* were enriched in the Ctrl and DHEA groups. Additionally, they were negatively correlated with the AUC-Glucose and FBG levels, and positively correlated with the TG levels. In contrast, CAG36 and CAG46 composed of ASVs from *Lachnospiraceae*, CAG38 composed of ASVs from *Blautia*, CAG47 composed of ASVs from *Desulfovibrionaceae* and *Blautia*, CAG54 composed of ASVs from *Alistipes* and *Muribaculaceae*, and CAG56 composed of ASVs from *Bacteroides* and *Alistipes* were enriched in the HFD and DHEA+HFD groups and showed an opposite trend in their correlations with the disease phenotypes (**Figures 3D, E** and **Table S1**).

### Different treatments induced a PCOS-like phenotype in pseudo germ-free mice

Quantitative real-time PCR and viable bacterial plate counts confirmed that more than 99.99% of the gut bacteria were depleted after 3 weeks of ABX treatment. These results verified the successful establishment of a pseudo germ-free mouse model.

**Figure 4:**
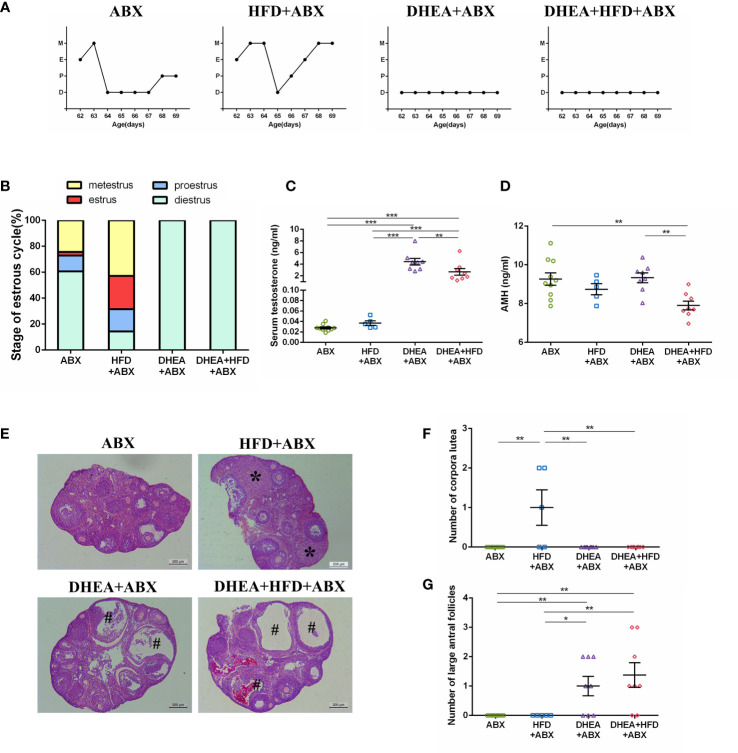
The stage of the oestrous cycle, ovarian morphology and serum T level in pseudo germ-free mice. **(A)** Representative oestrous cycle of one mouse from each group. **(B)** The proportion of each oestrous cycle stage in each group. **(C)** Serum testosterone level. **(D)** Serum AMH level. **(E)** Representative HE staining of ovarian tissue from one mouse from each group. * indicates corpora lutea, and # indicates large antral follicles. The number of **(F)** corpora lutea and **(G)** large antral follicles of one ovary from each group. Data are reported as the means ± SEMs. **P <0.01, and ***P < 0.001. AMH, anti-Mullerian hormone.

Similar to wild-type mice, all of the mice from the DHEA+ABX and DHEA + HFD+ABX groups displayed disrupted oestrous cycles, increased serum T levels, several antral follicles and no CL (**Figures 4A–G**). The AMH level in DHEA + HFD+ABX group was significantly lower than that of the ABX and DHEA+ABX groups (**Figure 4D**). These results suggest that DHEA can also induce the PCOS-like mouse model in pseudo germ-free mice. However, pseudo germ-free mice in the ABX group displayed a reduced oestrous stage and no CL (**Figures 4A, B, E, F**), indicating oligo- or anovulation in pseudo germ-free mice.

**Figure 5:**
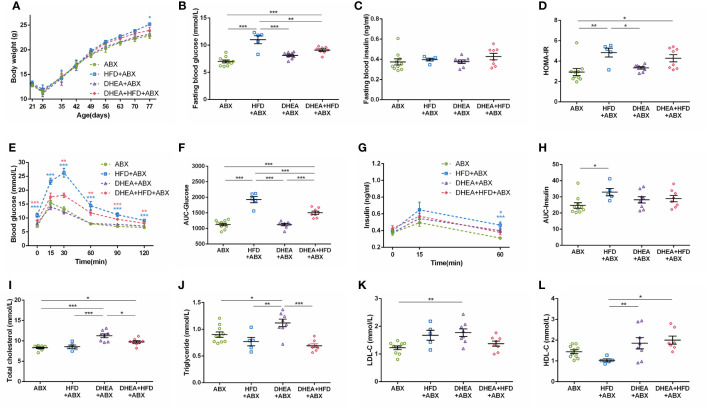
Glucolipid metabolism in pseudo germ-free mice. **(A)** Body weights. **(B)** Fasting blood glucose levels. **(C)** Fasting blood insulin levels. **(D)** HOMA-IR. **(E)** Serum glucose levels during the OGTT. **(F)** AUC-Glucose. **(G)** Serum insulin levels during the OGTT. **(H)** AUC-Insulin. **(I)** Total cholesterol. **(J)** Triglycerides. **(K)** LDL-C. **(L)** HDL-C. *P < 0.05, **P <0.01, and ***P < 0.001. HOMA-IR, homeostasis model assessment for insulin resistance index; AUC, area under the curve; LDL-C, low-density lipoprotein cholesterol; HDL-C, high-density lipoprotein cholesterol.

For all mice, body weights decreased progressively during the first week of ABX treatment and gradually recovered thereafter. At the end of the experiment, the body weight of the HFD+ABX group was significantly higher than that of the ABX group (**Figure 5A**). Similar to wild-type mice, the HFD+ABX and DHEA+HFD+ABX groups displayed markedly higher FBG levels (**Figure 5B**) and glucose intolerance (**Figures 5E, F**) than the ABX and DHEA+ABX groups. There was no significant difference in the FINS level among the four groups (**Figure 5C**). The AUC-Insulin level of the DHEA+ABX and DHEA+HFD+ABX groups was not significantly higher than that of the ABX group (**Figures 5G, H**). The HFD+ABX and DHEA+HFD+ABX groups displayed a significantly increased HOMA-IR index (**Figure 5D**). In addition, the TC, TG and LDL-C levels of the DHEA+ABX group were significantly higher than those of the ABX group, and the TC level of the DHEA+HFD+ABX group was significantly higher than that of the ABX group (**Figures 5I–L**).

### Effect of gut microbiota depletion on the phenotype of PCOS mouse models

**Figure 6:**
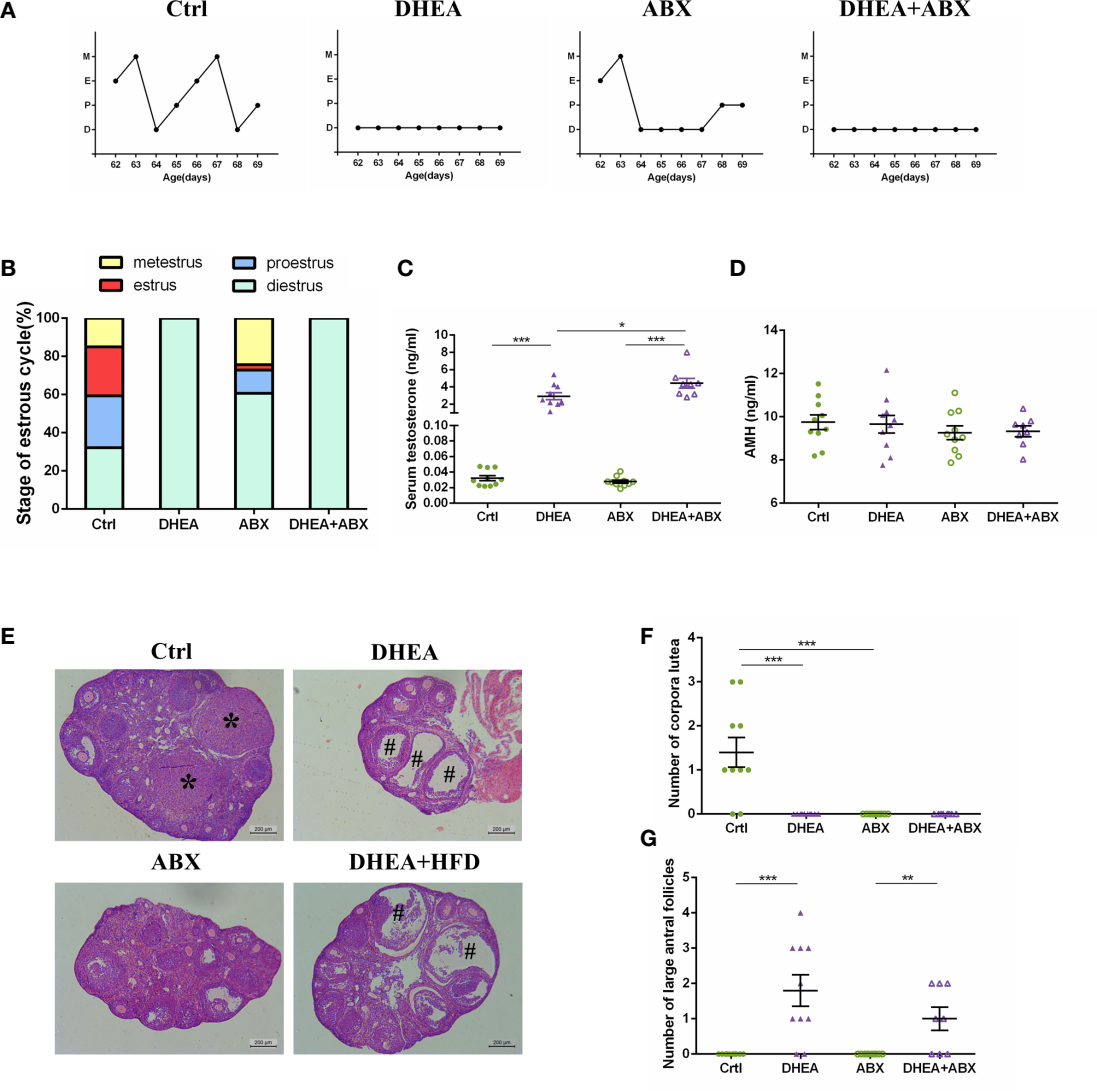
The stage of the oestrous cycle, ovarian morphology and serum T level of wild-type mice compared to pseudo germ-free mice. **(A)** Representative oestrous cycle of one mouse from each group. **(B)** The proportion of each oestrous cycle stage in each group. **(C)** Serum testosterone level. **(D)** Serum AMH level. **(E)** Representative HE staining of ovarian tissue from one mouse from each group. * indicates corpora lutea, and # indicates large antral follicles. The number of **(F)** corpora lutea and **(G)** large antral follicles of one ovary from each group. Data are reported as the means ± SEMs. **P <0.01, and ***P < 0.001. AMH, anti-Mullerian hormone.

To explore the effect of gut microbiota depletion on the phenotype of PCOS mouse models, we compared the reproductive endocrine and metabolism indicators of wild-type and pseudo germ-free mice. The disrupted oestrous cycles, AMH level, numbers of antral follicles and CL were not significantly different between the DHEA and DHEA+ABX groups (**Figures 6A, B, D–G**). In contrast, the DHEA+ABX group displayed significantly higher serum T levels than the DHEA group (**Figure 6C**).

**Figure 7:**
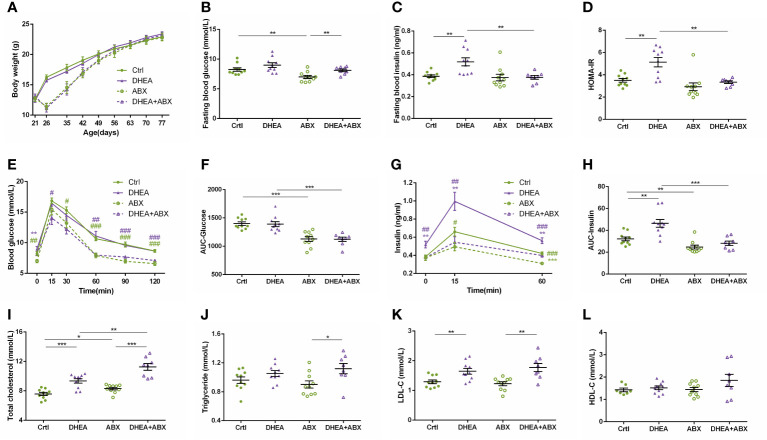
Glucolipid metabolism of wild-type mice compared to pseudo germ-free mice. **(A)** Body weights. **(B)** Fasting blood glucose levels. **(C)** Fasting blood insulin levels. **(D)** HOMA-IR. **(E)** Serum glucose levels during the OGTT. **(F)** AUC-Glucose. **(G)** Serum insulin levels during the OGTT. **(H)** AUC-Insulin. **(I)** Total cholesterol. **(J)** Triglycerides. **(K)** LDL-C. **(L)** HDL-C. *P < 0.05, **P <0.01, and ***P < 0.001. HOMA-IR, homeostasis model assessment for insulin resistance index; AUC, area under the curve; LDL-C, low-density lipoprotein cholesterol; HDL-C, high-density lipoprotein cholesterol.

The decrease in body weight induced by ABX treatment was restored to the level of the wild-type mice after 6 weeks, and there was no significant difference among the four groups at the end of the experiment (**Figure 7A**). The mice in the ABX and DHEA+ABX groups displayed markedly lower FBG levels (**Figure 7B**) and were less glucose-intolerant (**Figures 7E, F**) than the Ctrl and DHEA groups. Unlike the wild-type mice, the FINS, HOMA-IR, 15 min insulin and AUC-Insulin levels (**Figures 7C, D, G, H**) of the ABX and DHEA+ABX groups were not significantly different. In addition, the DHEA+ABX group displayed significantly higher TC levels and markedly higher TG levels than the other three groups (**Figures 7I–L**).

## Discussion

PCOS mouse models are often heterogeneous, especially in terms of metabolic features, but studies are still limited. Our current study first indicated that the gut microbiota was associated with heterogeneous phenotypes in PCOS mice. In this study, we induced PCOS in mice by DHEA administration or DHEA administration together with a HFD and ABX to observe the differences in reproductive and metabolic phenotypes. PCOS mice induced by DHEA alone showed reproductive disorders and hyperinsulinemia, and the HFD mainly aggravated glucolipid metabolic disorders. Glucolipid metabolic disorders were associated with alterations in the gut microbiota. The PCOS mice with gut microbiota depletion showed improvements in hyperinsulinemia and glucose metabolic disorders and more serious hyperandrogenism and lipid disorders.

In the past decade, the number of studies on PCOS has increased exponentially. A number of studies suggest that women with PCOS exhibit hyperandrogenism, multi-cystic ovaries, hyperinsulinemia, glucolipid metabolism disorders and obesity, which are related to the gut microbiota (14, 26, 27). However, women with PCOS often show high clinical heterogeneity, and our previous study found that the clinical heterogeneity of PCOS was related to the gut microbiota (18). Combined with reviews of PCOS rodent models over the past decades, we found that PCOS rodent models are often variable (9, 10). Lai et al. (11) used a HFD combined with DHEA to induce PCOS mouse models and found that HFD treatment did not affect the reproductive phenotype of DHEA-treated mice but caused significant metabolic alterations. Considering the effects of a HFD on metabolism in DHEA-treated mice, we treated C57BL/6 mice with DHEA alone or in combination with a HFD and studied the heterogeneity and its relationship with the gut microbiota.

Consistent with previous studies (5, 11), DHEA-treated and DHEA+HFD-treated PCOS mice showed disturbed cyclicity, multi-cystic ovaries and hyperandrogenism. Recently, Hohos et al. (28) found that mice fed a HFD for 10 weeks exhibited ovulatory dysfunction, which appears to be mediated through the dysregulation of ovarian Edn2 expression. In our study, HFD-fed mice did not show ovulatory dysfunction, and these conflicting results might be due to the time of HFD feeding. Thus, ovulatory dysfunction was caused by the effect of DHEA. It is well known that the HFD is frequently used to induce obesity, IR and hyperinsulinemia (29). However, we found that DHEA-treated mice showed hyperinsulinemia and IR, while DHEA+HFD-treated mice did not show this, except the two mice with extreme values. This may be due to the short duration of HFD treatment in our study. In addition, DHEA could lead to hyperinsulinemia by affecting granulosa cells (30). Our study suggested that DHEA could better induce PCOS in mice with hyperandrogenism and hyperinsulinemia than DHEA+HFD. Mice fed a 60% HFD showed impaired glucose tolerance, but the DHEA-treated mice showed normal glucose tolerance. The above result indicates that the impaired glucose tolerance was mainly induced by HFD. Therefore, we could conclude that the physiological and pathological pathways for reproductive and metabolic disorders in DHEA-induced PCOS mice may be different.

We found that the gut microbiota was altered in PCOS mice induced by DHEA or combined with HFD. Up to now, only one study reported that the relative abundance of *Lactobacillus* was lower in mice with prenatal androgen exposure, and no study reported the alteration of *Bifidobacterium* in PCOS mice (31). Our study showed the relative abundance of *Bifidobacterium* and *Lactobacillus* was significantly increased in the mice treated with DHEA, indicating the enrichment of *Bifidobacterium* and *Lactobacillus* by androgen exposure. Previous studies showed that *Bifidobacterium* and *Lactobacillus* could improve hyperandrogenism, obesity and IR of PCOS (32–34). However, our study showed the relative abundance of *Bifidobacterium* was positively correlated with the level of testosterone. These results suggest that the causal relationship between *Bifidobacterium* and androgen remains to be further explored. Moreover, a HFD might play a more critical role in shaping the gut microbiota structure than DHEA, emphasizing the important influence of diet on the host gut microbiota (35). Our study showed that the *Bacteroides*, *Blautia* and *Desulfovibrionaceae* specifically enriched in mice treated with HFD were positively correlated with the AUC-Glucose and FBG levels, while *Turicibacter* and *Lactobacillus* specifically inhibited in mice treated with HFD showed an opposite trend correlation with the disease phenotypes. Sun et al. (36) and Jiang et al. (16) pointed out that two strains of *Bacteroides* affected the host metabolic disorders and inflammation of host *via* the gut microbiota–bile acid axis. Lin et al. (37) reported an increase in the abundance of *Blautia* genera was correlated with the alterations of bile acids in rats treated with HFD. *Blautia* and *Desulfovibrionaceae*, the HFD-dependent taxa, were related to the glucose homeostasis (38). In this study, Zhao et al. also found that the enrichment of *Turicibacter* and *Bifidobacterium* was related to the improvement of glucose homeostasis. One analysis of the alteration of gut microbiota richness and structure in women with PCOS found that the dysbiosis of the gut microbiota was more severe in obese PCOS women than in nonobese women (17). These results suggest that changes in the gut microbiota mainly affected the metabolic disorders more than reproductive disorders by regulating bile acid metabolism and inflammation of the host.

In pseudo germ-free mice, DHEA treatment could still induce reproductive disorders. Han et al. (4) also induced PCOS in rats with disturbed cyclicity, multi-cystic ovaries and hyperandrogenism by treatment with DHEA+ABX. The above results indicated that the gut microbiota could not prevent the occurrence of reproductive disorders in PCOS rodent models induced by DHEA. The ABX-treated mice showed disturbed cyclicity and anovulation, while the HFD+ABX-treated mice showed normal ovulation. It is interesting that this phenomenon was associated with the change in adipose tissue weight (**Figure S2**). Serum AMH is the preferred ovarian reserve marker. PCOS rats showed significant decrease of AMH level in serum and increase of AMH protein expression in ovaries (39). In our study, serum AMH level was only significantly decreased in the DHEA+HFD+ABX group. However, the serum AMH level of the ABX-treated mice was not decreased, indicating the reproductive function is normal in pseudo germ-free mice. The underlying mechanisms require further investigation. AMH is an indicator that was tested four years later than the original experiment. Because of the long storage time and freeze-thawing of the samples, the result of AMH needs to be further verified. A Vietnamese study reported that lean PCOS women presented with anovulation, higher serum testosterone levels and a low metabolic disease risk (40). In a 2007 study, Martin et al. (41) found that caloric restriction led females become ceased cycling and underwent endocrine masculinization. However, until now, studies on lean PCOS are limited. Gut microbiota depletion did not prevent the glucose metabolic disorders induced by a HFD. Fleissner et al. (42) found that the absence of the gut microbiota in germ-free mice could not protect mice from diet-induced obesity. Metabolic disorders were not affected solely by the gut microbiota.

By comparing wild-type and pseudo germ-free mice, we found that gut microbiota depletion did not affect the occurrence of oestrous cycle disorders, serum AMH levels or ovarian morphological changes induced by DHEA but aggravated hyperandrogenism. Consistent with our results, Markle et al. found that the serum T level of germ-free female mice is significantly higher than that of SPF female mice and that the transplantation of the gut microbiota from male mice to germ-free female mice significantly increased the serum T level of the recipients (43). The serum T levels of PCOS mice in our study seemed to be negatively correlated with body fat weight. This phenomenon that might be due to the conversion of androgens to oestrogens by the aromatase enzyme that is expressed in fat tissue (44). These results suggest that the gut microbiota is not a direct factor in the development of hyperandrogenemia. The blood glucose level of PCOS mice was decreased after ABX intervention. ABX-induced gut microbiota depletion alters metabolic homeostasis by affecting colonic metabolism. Zarrinpar et al. (45) found that antibiotic-induced microbiome depletion decreased luminal *Firmicutes* and *Bacteroidetes* and thereby decreased luminal short-chain fatty acids (SCFAs). This was especially true for butyric acid, which can provide intestinal epithelial cells with energy. In addition, the body weight and parametrial fat weight of pseudo germ-free mice were lower than wild-type mice (**Figure S3**). Therefore, the decrease of blood glucose levels might correlate with emaciation. It suggested that we should pay attention to the effect of ABX on blood glucose of the host in future studies. ABX-induced gut microbiota depletion promoted glucose uptake in brown adipose tissue and the cecum (46). ABX-induced gut microbiota depletion could reduce the content of lipopolysaccharide in the ileum and inhibited the TLR4-related inflammatory pathways (47). Therefore, ABX treatment improved the chronic metabolic inflammation of the host. However, the PCOS mice treated with ABX showed more severe hyperlipemia. Although recent studies suggested that ABX could reduce lipid levels of HFD-fed mice, two studies reported the liver lipid accumulation by ABX treatment in tacrolimus-treated mice and the ABX discontinuity in db/db mice, which might be due to the reduced SCFAs and bile acid metabolism (48, 49). The specific mechanism for this still requires further study.

Our study focused on the correlational relationship between the gut microbiota and the heterogeneity of PCOS mouse models. Next, fecal microbiota transplantation of PCOS women to germ-free mice is needed to explore the causal relationship and mechanisms of the gut microbiota in the heterogeneity of PCOS.

Our work described the phenotypic differences in PCOS induced by different methods in mice and revealed that the phenotype of PCOS mice was correlated with the gut microbiota. Compared to DHEA+HFD, the PCOS mouse model induced by DHEA alone better simulates the stabilized pathophysiological defects of this disease. ABX intervention improved glucose metabolic disorders and hyperinsulinemia but aggravated hyperandrogenism and lipid metabolic disorders in PCOS mice. These findings provide new insight into the establishment of PCOS rodent models and studies about the pathophysiological mechanisms of PCOS in the future.

## Data availability statement

The raw sequence data presented in the study are deposited in the NCBI Sequence Read Archive database, accession number PRJNA672803.

## Ethics statement

The animal study was reviewed and approved by the Institutional Animal Care and Use Committee of Shanghai Jiaotong University (A2017062).

## Author contributions

XW performed the animal experiments, DNA extraction and sequencing, bioinformatics and statistical analysis. LG performed the animal experiment and statistical analysis. YZ and CX performed the animal experiments. YP designed the study and performed quality control. XD designed the study and wrote the manuscript. All authors contributed to the article and approved the submitted version.
