# Supplementary material for: Effects of dehydroepiandrosterone alone or in combination with a high-fat diet and antibiotic cocktail on the heterogeneous phenotypes of PCOS mouse models by regulating gut microbiota
Source: Front Endocrinol (Lausanne). 2022 Dec 22;13:1030151. doi: 10.3389/fendo.2022.1030151 (PMC9813398; doi:10.3389/fendo.2022.1030151)
Supplement: Supplementary file 1 [file DataSheet_1.docx]

***Supplementary Material***

This file includes:

Materials and Methods

Figs. S1 to S3

**Materials and Methods**

**Morphology**

After the collection of blood samples, parametrial fat tissues were removed and weighed and fixed in 4% paraformaldehyde immediately. The tissues were then embedded in paraffin and stained with haematoxylin and eosin (HE). Parametrial adipocyte cell size was analysed by Image-Pro Plus 6.0.

**Supplementary Figures**


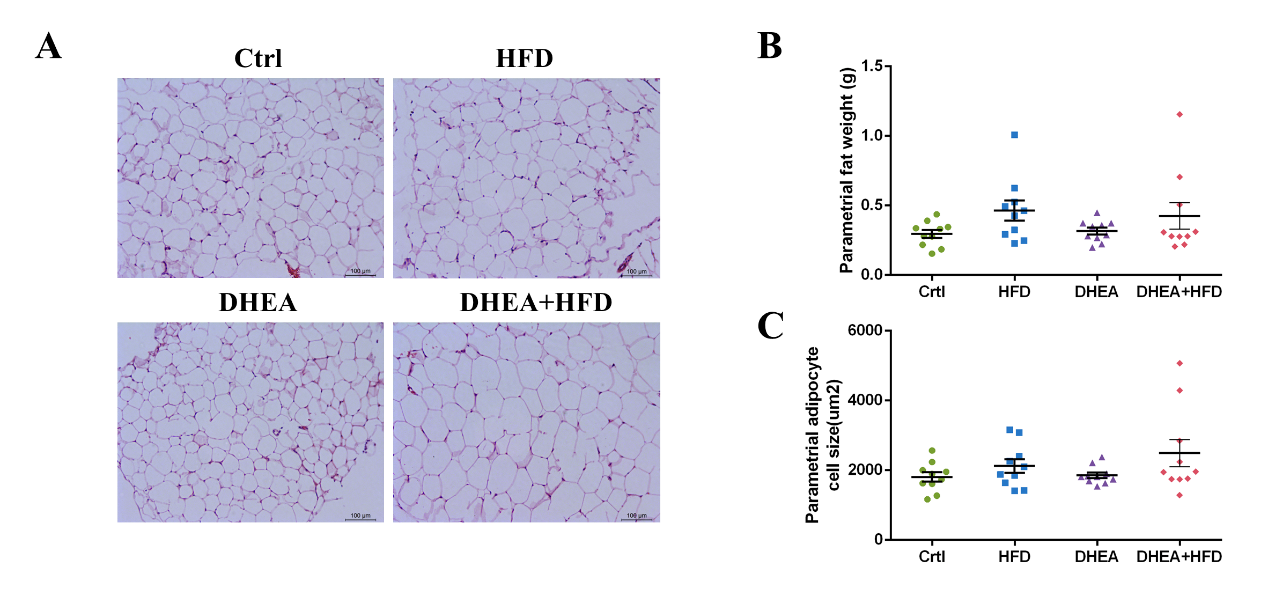


Figure S1. Parametrial fat weight and morphology in wild-type mice. (A) Representative HE staining of parametrial fat tissues from each group. (B) Parametrial fat weight. (C) Parametrial adipocyte cell size. Data are reported as means ± SEMs. There was no significant difference among the groups.


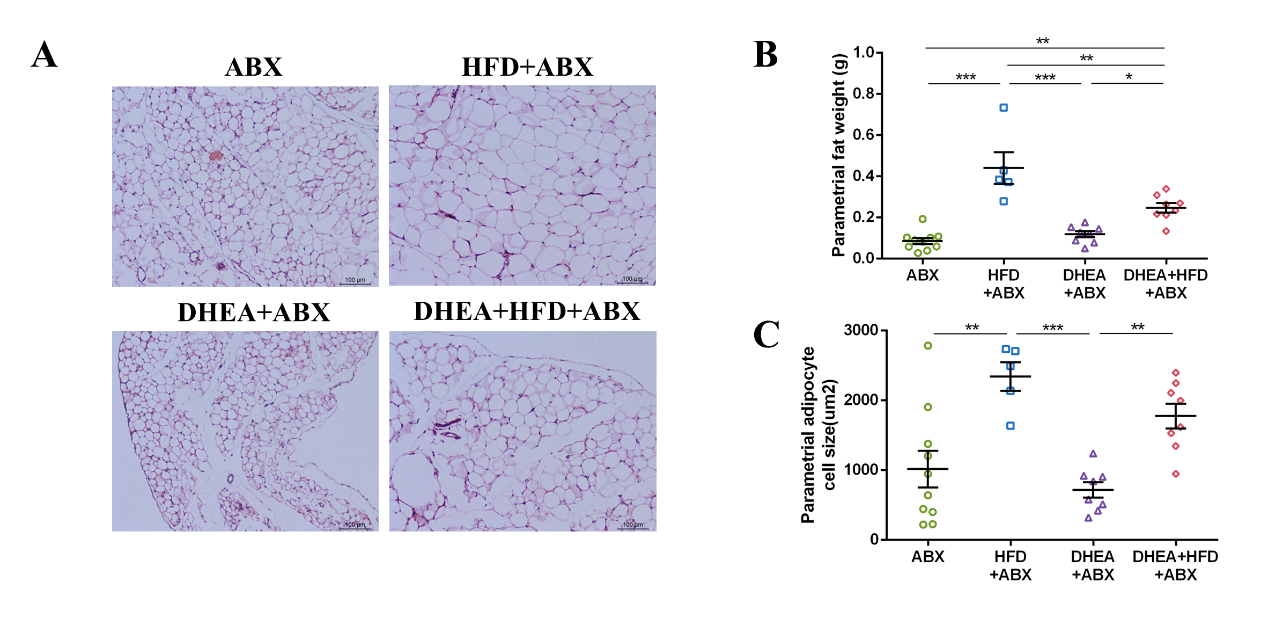


Figure S2. Parametrial fat weight and morphology in pseudo germ-free mice. (A) Representative HE staining of parametrial fat tissues from each group. (B) Parametrial fat weight. (C) Parametrial adipocyte cell size. Data are reported as means ± SEMs. **P* < 0.05, ***P* <0.01, and ****P* < 0.001.


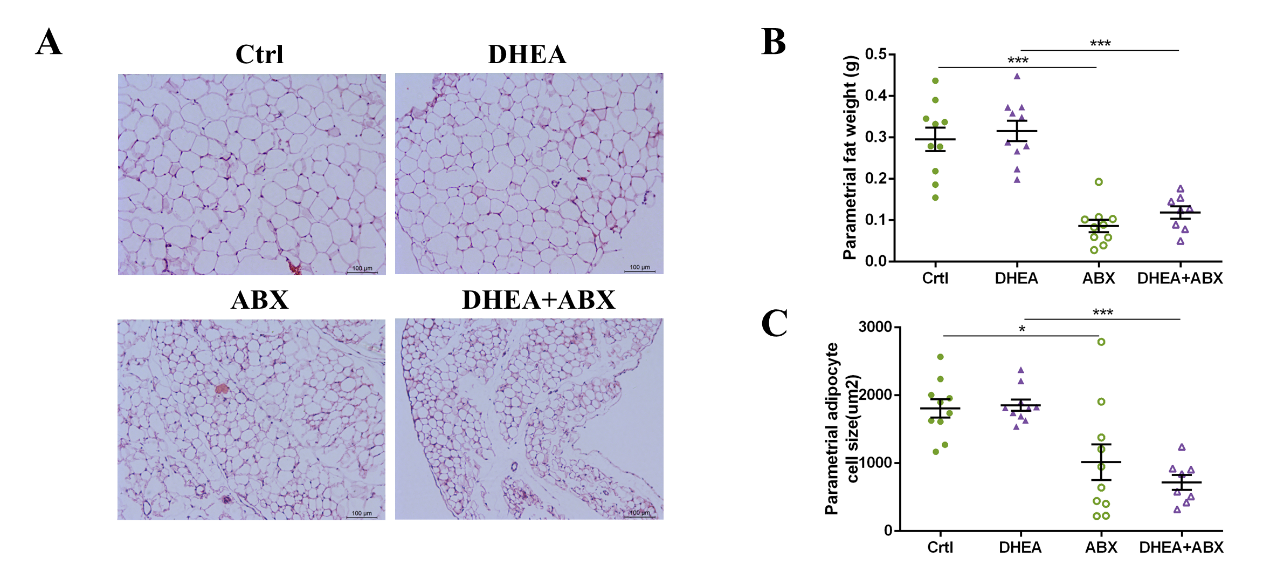


Figure S3. Parametrial fat weight and morphology of wild-type mice compared to pseudo germ-free mice. (A) Representative HE staining of parametrial fat tissues from each group. (B) Parametrial fat weight. (C) Parametrial adipocyte cell size. Data are reported as means ± SEMs. **P* < 0.05, and ****P* < 0.001.
